# Advanced diesel emission control in agricultural tractors using Ni-CNT_S_ nanocomposites and hybrid activated carbon–magnesium oxide adsorbents

**DOI:** 10.1038/s41598-025-24594-4

**Published:** 2025-11-18

**Authors:** Mayada E. Abdel Razek, Magdy A. Baiomy, A. Z. Taieb, Mohamed Refai, Gamal E. M. Nasr

**Affiliations:** 1 Mechanized Agriculture Sector, Agricultural Research Center, Ministry of Agriculture, Dokki, Giza, Egypt; 2 Bio-Engineering Department, Agricultural Engineering Research Institute, Agricultural Research Center, Dokki, Giza, Egypt; 3 Agricultural Engineering Department, Faculty of Agriculture, Cairo University, Giza, 12613 Egypt

**Keywords:** Diesel exhaust treatment, CNT nanocomposite filter, Field-based emission testing, Nanomaterials, Nickel–carbon nanotubes, Agricultural machinery, Engineering, Materials science

## Abstract

Agricultural tractors powered by thermally efficient and economically viable diesel engines play a pivotal role in the mechanization farming operations. However, these engines emit harmful pollutants such as carbon monoxide (CO), hydrocarbons (HC), nitrogen oxides (NO_x_), total suspended particles (TSP), and sulfur dioxide (SO_2_), all of which pose significant risks to human, animal, and plant health. In addition, these emissions also contribute to air pollution, global warming. This study builds upon our previous simulation-based research by implementing two novel exhaust gas treatment prototypes directly onto agricultural tractors under real-world field conditions. The first prototype was filled with activated carbon impregnated with magnesium oxides at a 7:0.5 ratio, whereas the second was coated with a nickel–carbon nanotube (Ni-CNTs) composite at a 0.2% concentration. Field experiments were conducted during plowing operations using a nine-shank chisel plow at a fixed depth, with gas measurements taken at intervals between 10 and 40 min. The Ni-CNTs-based prototype achieved superior adsorption efficiencies: 85.1% for CO, 55.21% for HC, 33.71% for TSP, 90.8% for NO_x_, and 76.1% for SO_2_. In comparison, the AC–MgO prototype achieved removal efficiencies of 84.68% for CO, 50.0% for HC, 25.0% for TSP, 87.24% for NO_x_, and 67.39% for SO_2_.These findings underscore the promising potential of nanomaterial-integrated systems—particularly Ni-CNTs—in enhancing diesel exhaust treatment performance and promoting environmentally sustainable agricultural machinery.

## Introduction

Agricultural tractors play a crucial role in mechanizing agricultural farming operations, with diesel engines serving as the primary source of mechanical power in the agricultural sector. These machines predominantly rely on diesel engines because of their high thermal efficiency and lower operating costs compared to gasoline engines, making them widely adopted in agriculture^1^. In Egypt, the number of agricultural tractors is estimated at approximately 133,500, with a total power output of about 6.19 million kW, according to the Central Agency for Public Mobilization and Statistics^2^. Despite the advantages of diesel engines in terms of energy efficiency, their use leads to the release of harmful emissions due to the combustion process, which—depending on temperature and other operating conditions—can increase nitrogen oxides while still emitting particulate matter, hydrocarbons, and other pollutants^3^.

The International Agency for Research on Cancer (IARC), part of the World Health Organization (WHO), classified diesel engine exhaust as carcinogenic to humans (Group 1) in 2012, and this classification was reaffirmed in the latest IARC Monograph Volume 138 (2025), underscoring the continued global urgency to mitigate exposure^4^. While agricultural machinery such as tractors improves productivity, its reliance on diesel engines still contributes to air pollution through the emission of harmful pollutants^5^. Diesel engines are widely used in various agricultural applications, including tractors, combine harvesters, irrigation pumps, and crop transportation vehicles. Nanomaterials (NMs), such as carbon nanotubes (CNTs), have been increasingly investigated for air pollution control due to their exceptional adsorption properties, stemming from their large surface area and unique structural characteristics. Unlike traditional filtration, which relies on physical sieving, adsorption captures pollutants through surface interactions. CNTs possess high porosity and small pore sizes, enabling adsorption efficiencies exceeding 99%^6^. Studies have highlighted that multi-walled carbon nanotubes (MWCNTs) possess remarkable electrical, mechanical, optical, and chemical properties, making them ideal for environmental applications, including emission control. Activated carbon (AC) is widely used in industrial applications as an adsorbent due to its high porosity, large surface area, and diverse surface chemistry^7^. AC is widely applied in many fields due to its low cost, high porosity, and suitable chemical stability^8^. Previous studies have reported that the adsorption capability of commercial AC for diesel exhaust pollutants can reach approximately 70% under controlled laboratory conditions, depending on factors such as pollutant type, temperature, and activated carbon pore characteristics^9^. AC derived from coconut shells has a higher efficiency, with over 86.4% NO_X_ removal^10^. Additionally, AC is among the most effective adsorbents for SO_2_, thanks to its high porosity, excellent thermal stability, and abundance of oxygen-containing groups^11^. These features enhance its adsorption of sulfur dioxide by increasing the number of active removal sites. Magnesium oxide (MgO) particles are also effective at adsorbing carbon dioxide. When chemically combined with activated carbon, they significantly improve its adsorption efficiency^12^. Modification of activated carbon with MgO enhances its pore structure and adsorption capacity, particularly for CO and HC^13^. The study emphasizes the importance of developing customized treatments for diesel engine emissions, as different pollutants require distinct reduction approaches. In diesel exhaust after treatment systems, efficiency may decrease over time due to factors such as material degradation, particulate build-up, and thermal cycling. Regular inspection and maintenance are therefore critical to sustain optimal pollutant removal performance, as also observed in our short-term field trials^14^. Based on previous studies, magnesium oxide (MgO) nanoparticles have shown great potential in enhancing diesel engine performance and reducing certain harmful emissions due to their well-ordered crystal structure, high thermal stability, and oxygen-carrying capacity. Research has reported improvements in brake thermal efficiency of up to 5% and reductions in CO and HC emissions by 8% and 10%, respectively, when MgO was applied. These findings support the integration of MgO into exhaust treatment systems to improve combustion efficiency and mitigate environmental impact^15^. Similarly, single-walled carbon nanotubes (SWCNT) have demonstrated to further enhance combustion efficiency, increase cylinder pressure, and reduce CO emissions more effectively than MgO, owing to their high surface area, thermal stability, and uniform dispersion^16^.

Furthermore, the use of fuel additives to mitigate emissions poses challenges, as they can alter engine performance and combustion dynamics, requiring further research. In conclusion, this study emphasizes the need for tailored solutions and continuous research to improve treatment methods and reduce the environmental and health risks associated with these pollutants. AC, with its high porosity and significant adsorption capacity, is widely used in industry for pollutant removal.

This study builds on our previous work published in Scientific Reports^17^, where the materials were initially tested using a diesel exhaust simulation system. In the present work, we advance beyond simulation by validating two novel prototypes directly on agricultural tractors under real plowing conditions. One prototype employs AC modified with MgO, while the other integrates Ni–CNT nanocoatings. The novelty of this study lies in bridging the gap between laboratory-based evaluations and field-scale applications, providing the first comparative assessment of these nanomaterials for agricultural diesel emission control.

## Materials and method

### Methodology

This study employed an applied experimental methodology that combined laboratory-based preparation with full-scale field validation. Two exhaust treatment prototypes were developed: the first using activated carbon impregnated with magnesium oxide (AC–MgO), and the second coated with a nickel–carbon nanotube (Ni–CNTs) composite. The units were mounted on a Kubota M-100 agricultural tractor and tested under real plowing operations. Exhaust gas components (CO, HC, NO_x_, SO_2_, and TSP) were measured before and after prototype installation using calibrated instruments in accordance with ISO 8178 standards. Reliability was ensured through pre-test calibration, repeated measurements at multiple time intervals, and operation under uncontrolled field conditions to capture realistic variability. In addition, the filtration media were subjected to SEM and EDX analyses to characterize morphological and chemical changes after operation. Thermal performance and cost analyses were also conducted to evaluate the overall applicability of the prototypes. This integrated methodology allowed for a comprehensive assessment of emission reduction efficiency, thermal effects, and economic feasibility under practical agricultural conditions.

**Fig. 1:**
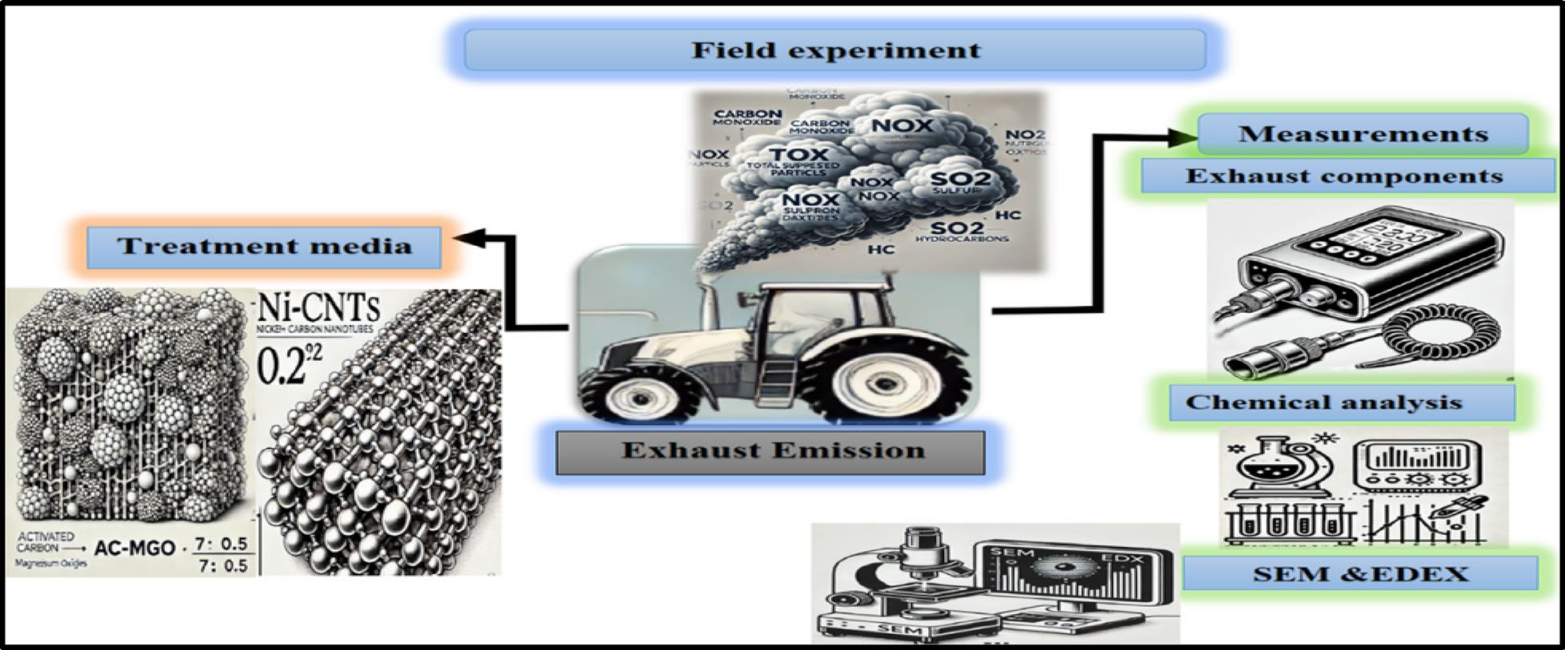
The setup of the experiment.

Two types of units were created: one with AC impregnated with MgO and another with a Ni-CNTs composite. These units were installed in the tractor’s exhaust pipe and tested. Emission components, including CO, HC, NO_x_, TSP, and SO_2_, were measured to evaluate the performance of each filtration unit. Figure 1 shows the step of the experiment.

### Prototypes for tractor exhaust treatment

**(a) First prototype: activated carbon-impregnated magnesium oxide unit**

**Fig. 2:**
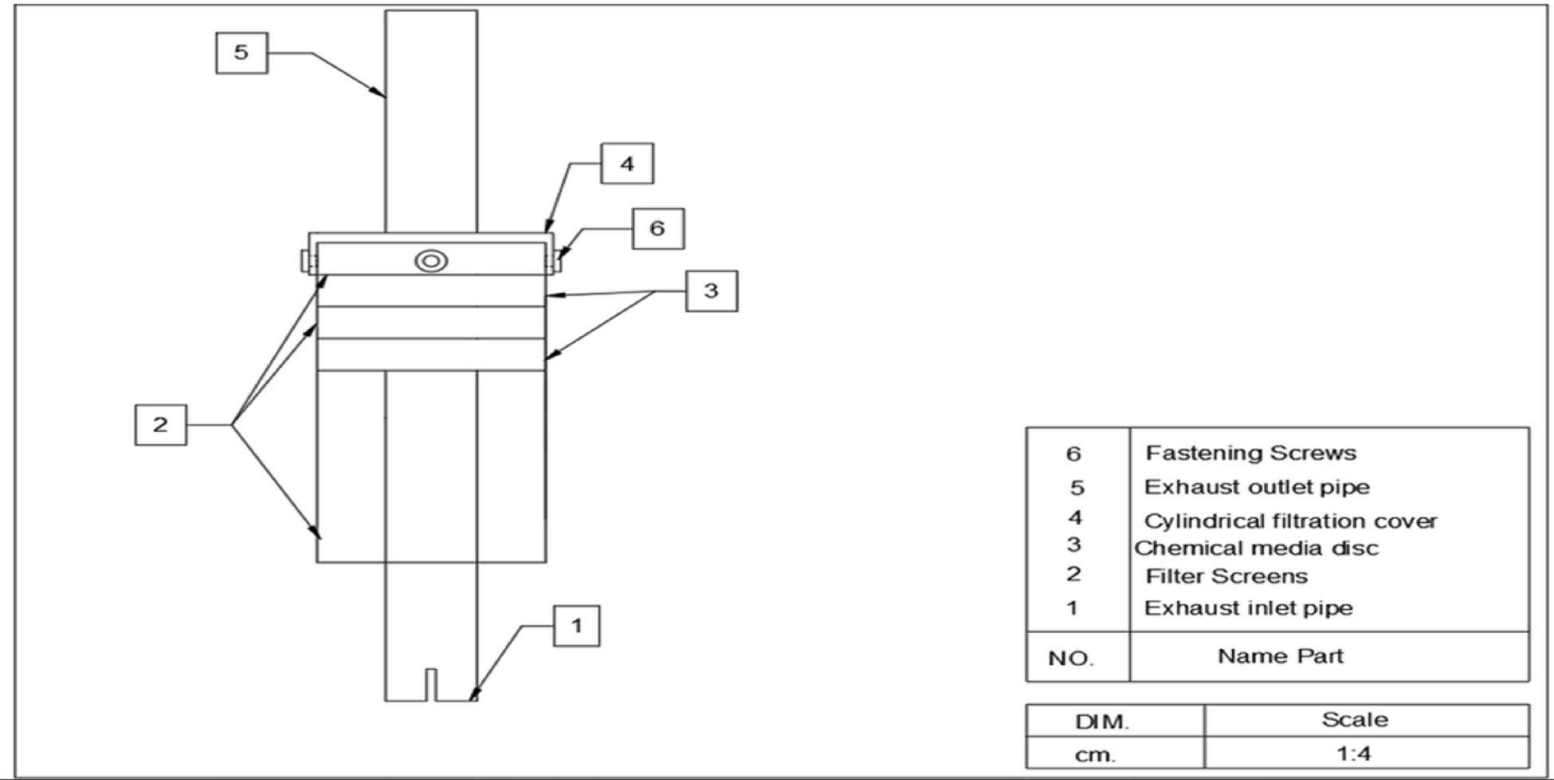
Components of First prototype.

The exhaust emissions treatment system includes an AC unit impregnated with MgO to reduce the amount of pollutants in tractor gases. The unit consists of:

- Treatment unit: A cylindrical filtration unit constructed from 1 mm thick sheet metal, measuring 30 cm in length and 14.4 cm in outer diameter.
- Exhaust inlet pipe: welded to the center of the bottom cover of the treatment unit, with dimensions of 17 cm in length and 65 mm in diameter to match the tractor’s exhaust pipe.
- Treatment unit cover: 3 cm in height and 145 mm in diameter, constructed from 1 mm thick sheet metal, with a centrally located hole of 65 mm in diameter.
- Pipe outlet: a conduit that carries the filtered exhaust gases out of the treatment unit, measuring 65 mm in diameter and 30 cm in length, welded in the central hole of the unit cover are shown in Fig. 2.
- Inside the treatment chamber, the chemical media discs serve as the primary adsorbent holders. Each disc is made from 1.2 mm thick sheet metal, measuring 3 cm in height and 13.5 cm in diameter. The discs are covered on both sides with stainless-steel 100 mesh screens, which act as a support structure to hold the adsorbent material (activated carbon impregnated with MgO or Ni-CNTs coating) while allowing exhaust gases to pass through. The discs ensure uniform gas–adsorbent contact, enhancing pollutant capture efficiency.

**Fig. 3:**
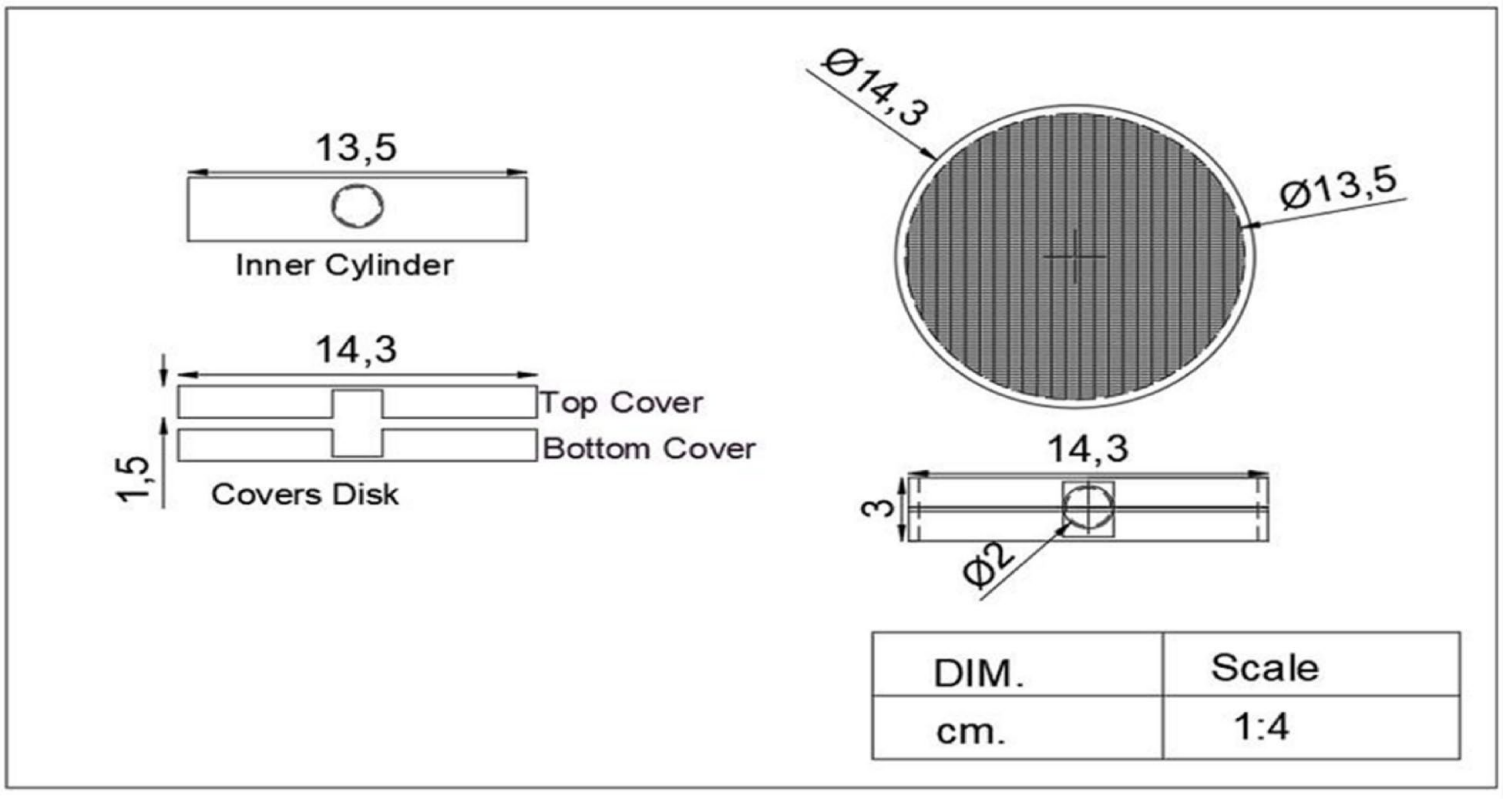
The chemical media disc.

Likewise, it contains a feeding hole with a diameter of 1.2 mm. An upper and lower cover are fabricated from sheet metal 1.2 mm with a height of 1.5 cm and a diameter of 14.3 cm for each. They are covered on the top and bottom by astainless-steel 100 mesh screen Fig. 3 shows the chemical media disc in the treatment unit.

**(b) Second prototype: Ni-CNTS unit**

**Fig. 4:**
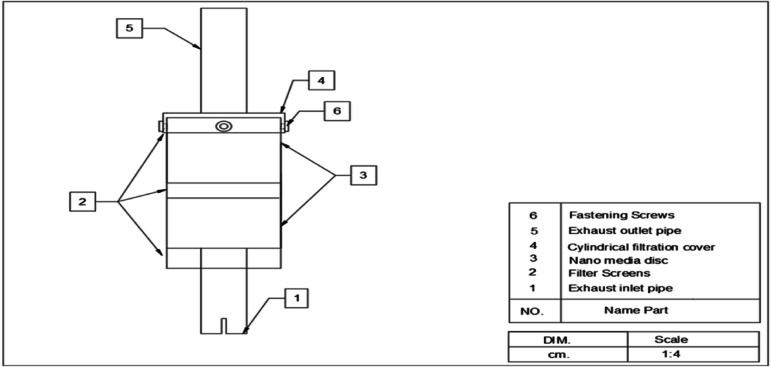
Components of Second Prototype.

**Fig. 5:**
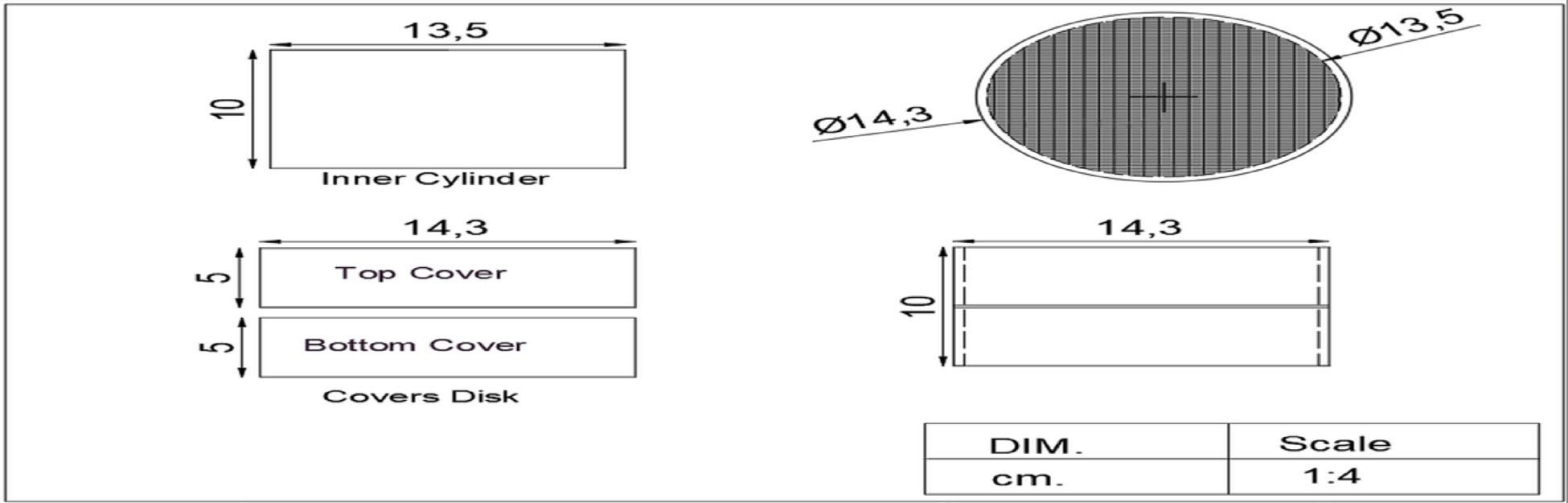
The Ni-CNTS media disc.

The exhaust emissions treatment system includes a Nano carbon unit to reduce the amount of pollutants in tractor exhaust gases. The unit consists of:

- The treatment unit, Exhaust Inlet Pipe, treatment Unit Cover, and Pipe Outlet were the same fabrication and components of the first unit (an active carbon unit). Figure 4 depicts the components and sections of the Ni-CNTS unit.
- The Nano media disc it was fabricated of sheet metal 1.2 mm thick. It is an inner disc with a height of 10 cm and a diameter of 135 mm. An upper and lower cover are manufactured with a height of 5 cm for each of them and a diameter of 144 mm. It is covered on the bottom and top with a sheet of stainless-steel 100 mesh screen Fig. 5 shows The Nano media disc in the treatment unit.

### The specifications of the tractor under test

Kubota M-100 tractor was chosen due to its large number in the agricultural engineering sector. This tractor is a critical component in modern farming operations, serving as a versatile workhorse for various tasks such as plowing, planting, cultivating, and transporting agricultural produce. Tests were conducted on the tractor under study in the Mechanized Agriculture Sector to implement the practical part of this study. Based on data collection, the Mechanized Agriculture Sector had 1483 Kubota tractors, and the experiment took place at the Qalyoub Station in Qalyoub Governorate, located in the Delta region of Egypt. The Kubota M-100 used in this study is powered by a four-cylinder, water-cooled diesel engine rated at 98.7 hp (73.6 kW) and certified to EPA Tier 1 emission standards, without advanced after-treatment systems. Table 1 shows the specifications of the tractor under test.

**Table 1:** The specifications of the tractor under test.

Specification	Value
Manufacturer	Kubota M-100
Type	Utility tractor
Engine Power	98.7 hp (73.6 kW)
Engine Type	Turbocharged diesel, 4-cylinder, liquid-cooled
Fuel System	Direct injection
Rated Engine RPM	2400 RPM
Fuel Capacity	52.8 gallons (199.8 L)
Fuel type	Diesel

#### Chisel plow specifications

The nine-shank mounted chisel plow features robust blades designed to penetrate soil depths from 15 to 45 cm, with a working width of 2—3 m. Built with a sturdy stainless steel frame and adjustable spacing, it includes a hydraulic control system for precise depth adjustment. The plow improves soil aeration, breaks up compacted layers, and enhances root development and water efficiency in difficult soil conditions.

### Location of the study area

Initial experiments were conducted using a diesel exhaust simulation unit at the Agricultural Engineering Research Institute—Agriculture Research Center. This simulation unit consists of a stationary diesel engine connected to a controlled exhaust flow and measurement system, enabling preliminary testing of filtration materials under repeatable laboratory conditions before final field experiments^17^. Purification materials under study were tested using this prototype. The final prototype, which included two filtration and treatment units. The two units were tried and tested on an M100 Kubota tractor, belonging to the mechanized agricultural sector, during the plowing of clay soil in Qalyubia Governorate using a nine-shank chisel plow.

#### Test repetitions and statistical considerations

This study was a full-scale field experiment on an M-100 Kubota tractor during actual plowing operations on clay soil in Qalyubia Governorate under real, uncontrolled environmental conditions. During field tests, the clay soil had an average moisture content of about 22%, with a terrain slope of approximately 1–2%. Ambient temperature ranged from 26 to 30 °C under partly cloudy conditions, reflecting typical spring weather in the region. Due to the continuous commercial-scale work, replicating identical trials was not feasible. Instead, reliability was ensured through continuous exhaust monitoring, pre-test calibration of all devices, and measurement recording at multiple time intervals within the same testing session. Natural variability in these real operating conditions was considered a more representative indicator of practical performance than controlled laboratory repetitions.

According to the 2021 Agricultural Machinery and Tractors Bulletin issued by the Egyptian Ministry of Agriculture and Land Reclamation, Egypt has approximately 133,500 agricultural tractors. This context highlights the significance of improving emission control technologies for widespread application in the national tractor fleet. In the Central Delta region, the number of agricultural tractors was reported as 418,040 units. Specifically, the number of Kubota tractors across Egypt was 18,254 units; in the Central Delta region, Kubota tractors accounted for 2936 units. These figures highlight the significant presence of mechanized farming in the region, emphasizing the role of tractors in enhancing agricultural productivity^18^.

### Characterization tests

**(a) Components of combustion exhaust**

Emission measurements were taken for a Kubota M100 tractor under various operating conditions (without load, Plow installation without load and plowing installation at a depth15cm). Levels of (CO), (TSP), (NOx), (HC), and (SO_2_) were measured. Subsequently, filtration units (AC impregnated with MgO and Ni-CNTS unit) were installed. The process generating the highest emission levels was identified as plowing at a depth of 15 cm. Emission components were measured before and after installing the filtration units using the Zhongan S360 device, and temperatures were recorded using the Thermometer model 1312-EN-01. All gas analyzers were calibrated prior to testing following ISO 8178 standards, with sensitivity checks performed at 0.1 ppm for CO and HC, and 0.5 ppm for NOx and SO_2_. Calibration intervals were maintained in accordance with the manufacturer’s recommendations. The adsorption efficiency was computed by comparing the initial gas emission with the final gas concentration. The adsorption efficiency can be computed by comparing the initial gas emission with the final gas concentration.

**(b) Chemical analysis**

The filtration unit, consisting of AC impregnated with MgO at a ratio of 7:0.5 underwent chemical analyses to determine the percentages of nitrates, sulfates, and carbonates before and after the treatment process using two devices: a Kjeldahl (BOCHI-320,China) and a spectrophotometer, V730, China at the Central Laboratory Network (CLN), National Research Center.

**(c) SEM and EDX analysis**

The filtration media, consisting of activated carbon impregnated with magnesium oxides (AC + MgO, 7:0.5) and Ni–CNTs (0.2%), were removed from the tractor-mounted units after field operation and analyzed using scanning electron microscopy (SEM) and energy-dispersive X-ray spectroscopy (EDX). SEM provided detailed images of the surface morphology and structural characteristics, while EDX identified the elemental composition and distribution on the media surface. All analyses were conducted at the National Research Center—Center of Excellence for Medical Research.

**(d) Thermal performance analysis of the filtration units**

The thermal performance of two exhaust gas filtration units—Prototype 1 (AC impregnated with MgO) and Prototype 2 (Ni-CNTs coated unit)—was evaluated to assess their impact on exhaust temperature. Heat dissipation and convective heat transfer coefficients were calculated using fundamental heat transfer equations, incorporating a constant exhaust gas mass flow rate and the specific heat capacity of the exhaust gases. The temperature drop across each filtration unit was measured to determine the thermal energy loss and heat transfer efficiency. The study considered the influence of material composition and structural properties on thermal dissipation, highlighting the role of adsorption and energy redistribution in enhancing the overall performance of the filtration system.

**(e) Cost analysis**

The comprehensive cost analysis for developing the final prototypes—Prototype 1 AC unit impregnated with MgO and Prototype 2 (Ni-CNTs unit)—included all associated MgO oxides, calculated per gram in Egyptian pounds (LE/g). Additionally, the analysis accounted for manufacturing and assembly costs of filtration discs, nano-coating stainless steel sheets, and preparation of the nickel plating bath, with plating bath costs calculated per liter (LE/L) or per gram ($/g). The cost of nanomaterials (NMS) required for Prototype 2 was also included.

To determine the total cost of each prototype, the formula was employed^19^:

1 $${\text{Initial Cost}}\left( {\text{I}} \right) = \left( {{\text{MU}}} \right) + \left( {{\text{MD}}} \right) + \left( {\text{S}} \right) + \left( {{\text{FM}}} \right) + \left( {\text{C}} \right)$$

where (MU) Manufacturing cost of the unit (LE) ($), (MD): Manufacturing cost of treated discs (LE) ($), (S): cost of stainless steel sheet (LE/m^2^) ($), (FM) Cost of filtration materials (LE/g) ($), (C): Cost of (Chemical (LE/g)/bath coating LE/L) ($).

The total cost calculation combines both fixed and variable costs, with fixed costs covering prototype and disc manufacturing, and variable costs including purification materials such as NM_S_, nano-coating bath, AC, and MgO. This can be expressed as:

2 $${\text{Total cost}} = {\text{Fixed costs}} + {\text{Variable costs}}$$

## Results and discussion

### Components of combustion exhaust

**(a) First prototype**

The exhaust emissions of an M-100 Kubota tractor were measured during plowing at a depth of 15 cm using a chisel plow with 9 tines. Exhaust temperature increased from 210 to 283 °C before unit installation, and decreased to 201–262 °C after installing the activated carbon unit impregnated with magnesium oxide. An adsorption efficiency of 84.68% was achieved for CO, with concentrations reduced from 248 to 38 mg/m^3^. Hydrocarbon removal efficiency was measured at 50%, with levels declining from 96 to 49 mg/m^3^. Total suspended particles were reduced by 25%, dropping from 28 to 21 mg/m^3^. The system recorded 87.24% efficiency for NOx, lowering concentrations from 196 to 25 mg/m^3^. A reduction of 67.39% was observed for SO_2_, with levels decreasing from 46 to 15 mg/m^3^.

The results of testing the activated carbon unit impregnated with magnesium oxide demonstrated the adsorption efficiency for each of the exhaust components as follows:

- (CO): An adsorption efficiency of 84.68% was achieved, with the concentration reduced from 248 to 38 mg/m^3^.
- (HC): The efficiency of hydrocarbon adsorption was 50%, with the concentration decreasing from 96 mg/m^3^ before filtration to 49 mg/m^3^ after the filtration process.
- (TSP): Filtration reduced the total suspended particles by 25%, with the concentration decreasing from 28 mg/m^3^ before filtration to 21 mg/m^3^ after filtration.
- (NO_x_): The system recorded an efficiency of 87.24%, lowering concentrations from 196 to 25 mg/m^3^.
- (SO_2_): A reduction of 67.39% was observed, with levels dropping from to 15 mg/m^3^.

These results can be attributed to the high porosity and adsorption capacity of activated carbon, enhanced by the incorporation of magnesium oxide. Magnesium oxide acts as a promoter, improving the interaction between the activated carbon and the pollutants, particularly nitrogen oxides and carbon monoxide, which are typically more difficult to capture. Additionally, the high surface area of the modified carbon material allows for a more efficient removal of various exhaust components, leading to improved overall filtration performance. These findings align with previous studies that highlight the effectiveness of activated carbon and its modifications in reducing harmful emissions from diesel engines^20,21^**.**

**(b) Second prototype**

During plowing with a 9-tooth chisel plow, exhaust emissions were measured for the M-100 Kubota tractor. Exhaust temperature increased from 210 to 283 °C before installation of the unit, with no significant change after installing the Ni-CNTs nanocomposite coating unit.An adsorption efficiency of 85.1% was achieved for CO, reducing concentrations from 248 to 37 mg/m^3^. HC removal efficiency reached 55.21%, with levels falling from 96 to 43 mg/m^3^. TSP levels dropped by 33.71%, from 28 to 18 mg/m^3^. The unit achieved a reduction of 90.8% for NOx, lowering concentrations from 196 to 18 mg/m^3^. Removal efficiency for SO_2_ was measured at 76.1%, with levels decreasing from 46 to 11 mg/m^3^.

The results of testing the Ni-CNTs nanocomposite coating unit demonstrated the adsorption efficiency for each of the exhaust components as follows:

- (CO): An adsorption efficiency of 85.1% was achieved, with the concentration reduced from 248 to 37 mg/m^3^.
- (HC): The efficiency of hydrocarbon adsorption was 55.21%, with the concentration decreasing from 96 mg/m^3^ before filtration to 43 mg/m^3^ after the filtration process.
- (TSP): Filtration reduced the total suspended particles by 33.71%, with the concentration decreasing from 28 mg/m^3^ before filtration to 18 mg/m^3^ after filtration.
- (NO_x_): The unit achieved a reduction of 90.8%, lowering concentrations from 196 to 18 mg/m^3^.
- (SO_2_): Removal efficiency was measured at 76.1%, with levels dropping from 46 to 11 mg/m^3^.

These results can be attributed to the unique properties of CNTs, including their high surface area, excellent electrical conductivity, and strong adsorption capability^22^. CNTs form a highly porous structure that enhances the interaction with exhaust gases, allowing for efficient pollutant capture. The exceptional performance in NO_x_ removal, in particular, can be linked to the ability of CNTs to facilitate chemical interactions with nitrogen oxides, a feature that has been widely documented in previous studies^23,24^**.** The high efficiency in capturing SO_2_ and CO can also be attributed to the enhanced chemical reactivity of the Ni-CNTs composite, which increases the adsorption sites and improves overall filtration efficiency^25^. This study’s findings support the use of CNT-based nanocomposites as a promising solution for diesel engine emission control, offering improved performance over traditional filtration systems. As detailed in the Methods section, identical repetitions under exactly the same field conditions were not feasible. Reported results therefore represent the average of multiple time-interval measurements within the same operational session, reflecting real-world variability.

**Fig. 6:**
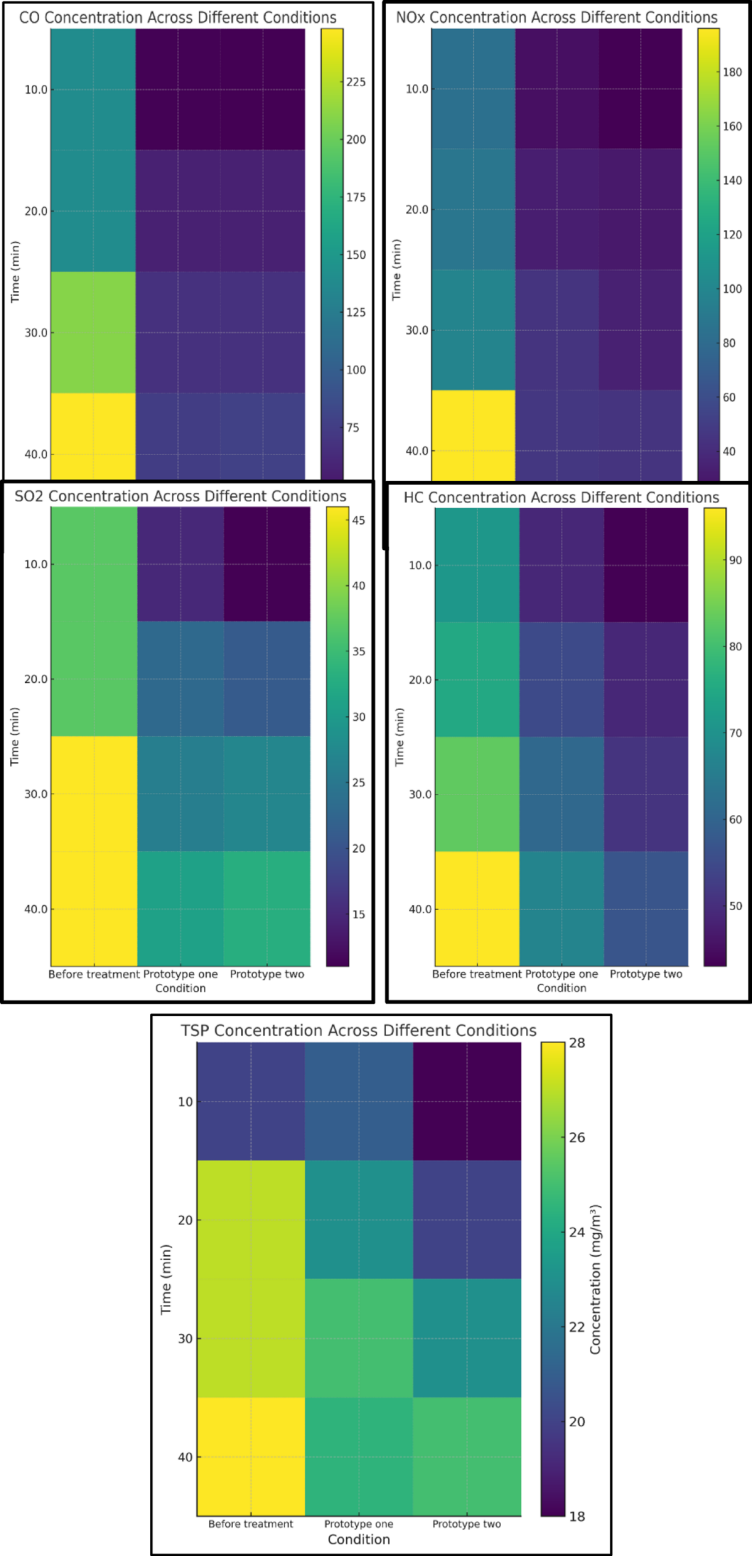
Comparison of Gas Emissions Before and After Treatment Using first prototype and second prototype.

Figure 6 presents a quantitative comparison of the emissions of major gases, including (NO_x_), (SO_x_), (CO), (TSP) and (HC), before and after treatment using first Prototype (Ni-CNTs) and second Prototype (AC-MgO). The results demonstrate a significant reduction in pollutant concentrations following the purification process, highlighting the effectiveness of both systems in improving exhaust quality. Furthermore, the comparison reveals variations in the performance of each prototype in pollutant removal, reflecting the influence of the physicochemical properties of the adsorbent materials on purification efficiency.

#### Long-term performance considerations

While the AC–MgO and Ni–CNTs prototypes demonstrated high adsorption efficiencies under the tested field conditions, the long-term performance of both systems is expected to be influenced by particulate matter accumulation (soot loading) on the adsorbent surfaces. Progressive deposition of diesel particulate matter can block micropores and reduce the effective surface area, leading to decreased adsorption capacity over time. This pore blockage may particularly affect the capture of gaseous pollutants, as the mass transfer pathways become restricted. To maintain efficiency, periodic cleaning or regeneration of the adsorbent media is recommended. Future work should include extended-duration field trials to evaluate the rate of performance decline and determine optimal maintenance intervals for sustainable operation.

**Fig. 7:**
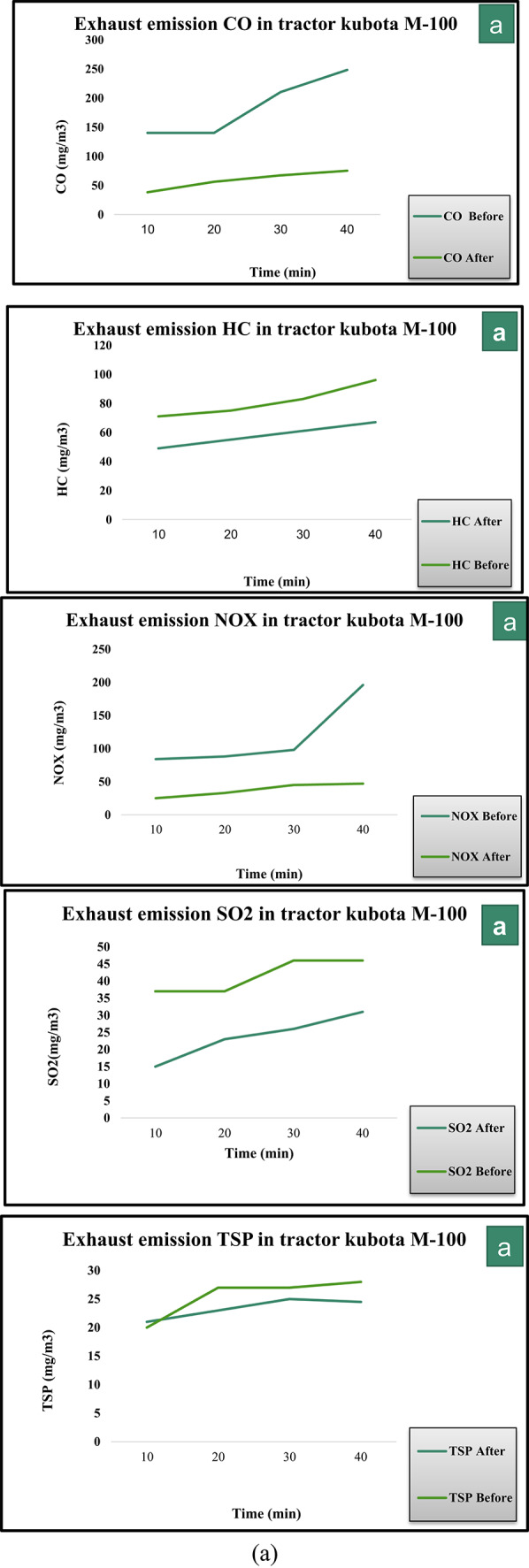

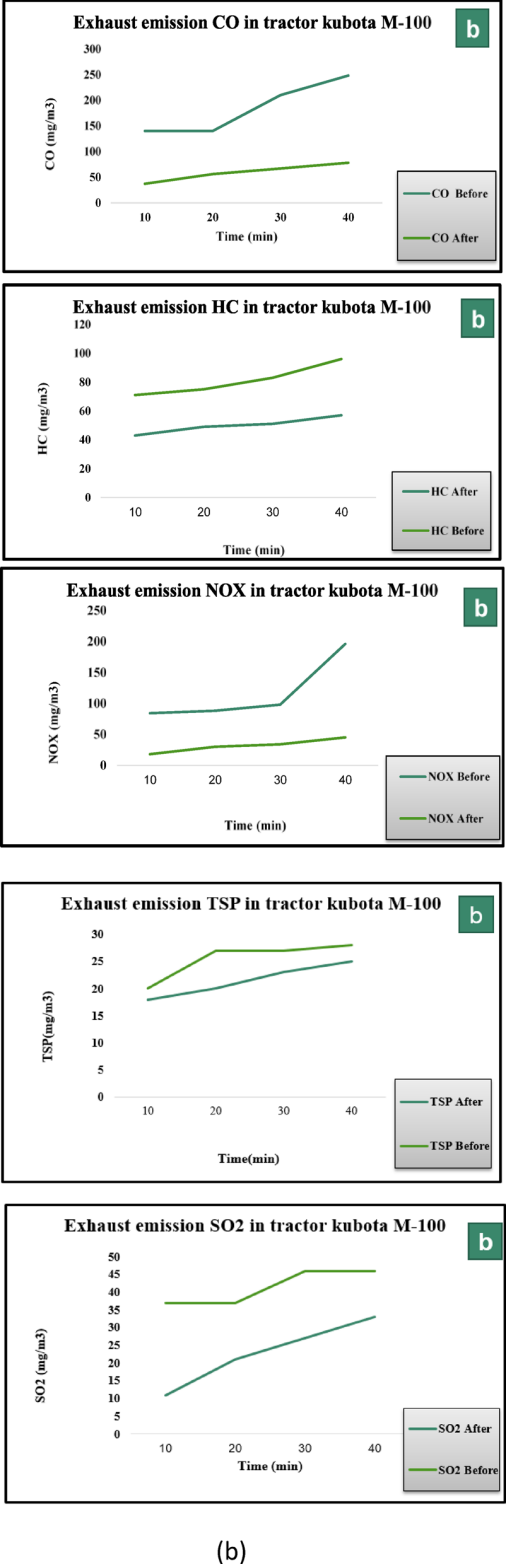
(**a**) Concentrations of CO, HC, NO_x_, SO_2_, and TSP before and after treatment using the AC–MgO prototype. (**b**) Concentrations of CO, HC, NO_x_, SO_2_, and TSP before and after treatment using the Ni–CNTs prototype.

Figure 7a and b illustrates the concentrations of key exhaust gas components—CO, HC, NO_x_, SO_2_, and TSP—before and after treatment using the AC–MgO and Ni–CNTs prototypes. The comparison clearly shows a reduction in all measured pollutants for both prototypes, with the Ni–CNTs unit exhibiting higher overall removal efficiencies, particularly for NO_x_ and SO_2_.

### Chemical analysis of first prototype

**Fig. 8:**
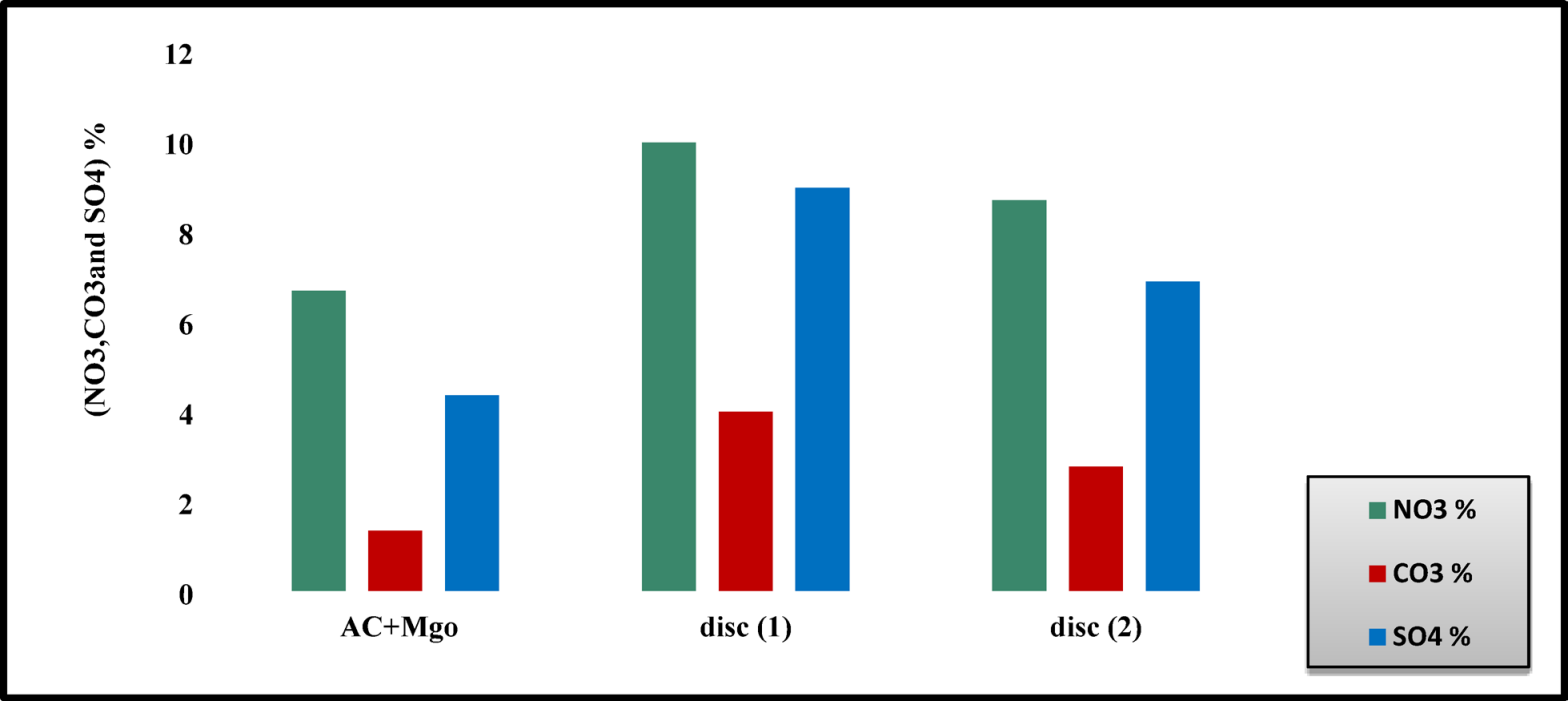
The percentages of carbonates, sulfates, and nitrates in the filter discs before (AC + Mgo) and after adsorption (disc 1&2).

Two filtration discs were chemically analyzed to quantify nitrates, carbonates, and sulfates, serving as indicators of nitrogen, carbon, and sulfur oxides before and after the filtration process Fig. 8. The increase in these compounds after treatment with MgO-enhanced activated carbon is attributed to chemical reactions catalyzed by magnesium oxide, coupled with the high surface area and basicity of the material. These properties enhance interaction with acidic pollutants such as NO_x_ and SO_2_, improving fixation efficiency. The first disc showed higher accumulation of nitrates, sulfates, and carbonates than the second disc, due to direct exposure to higher pollutant concentrations at the engine outlet. By the time exhaust gases reached the second disc, pollutant levels had already declined, resulting in lower adsorption rates.

Figure 8 the labels disc (1) and disc (2) denote the sequential adsorption discs incorporated into the after-treatment prototype. In particular, disc (1) corresponds to the first disc that directly encounters the exhaust gases, while disc (2) refers to the second disc positioned downstream along the exhaust flow. This arrangement was deliberately designed to assess the progressive adsorption efficiency across multiple discs within the system. The increase in nitrates, sulfates, and carbonates in magnesium oxide-enhanced activated carbon is due to chemical reactions catalyzed by magnesium oxides, the large surface area of activated carbon, and the basic properties of magnesium oxide, which make the surface more reactive with acidic pollutants like NO_x_ and SO_2_. These factors enhance the adsorption and fixation efficiency of these compounds. The increase in sulfates, carbonates, and nitrates in the first disk compared to the second is due to its direct exposure to higher pollutant concentrations as they exit the engine, enhancing chemical reactions and the formation of these compounds. Additionally, pollutant concentrations are lower by the time they reach the second disk, reducing its rate of adsorption.

### SEM and EDX analysis

**(a) First prototype**

**Fig. 9:**
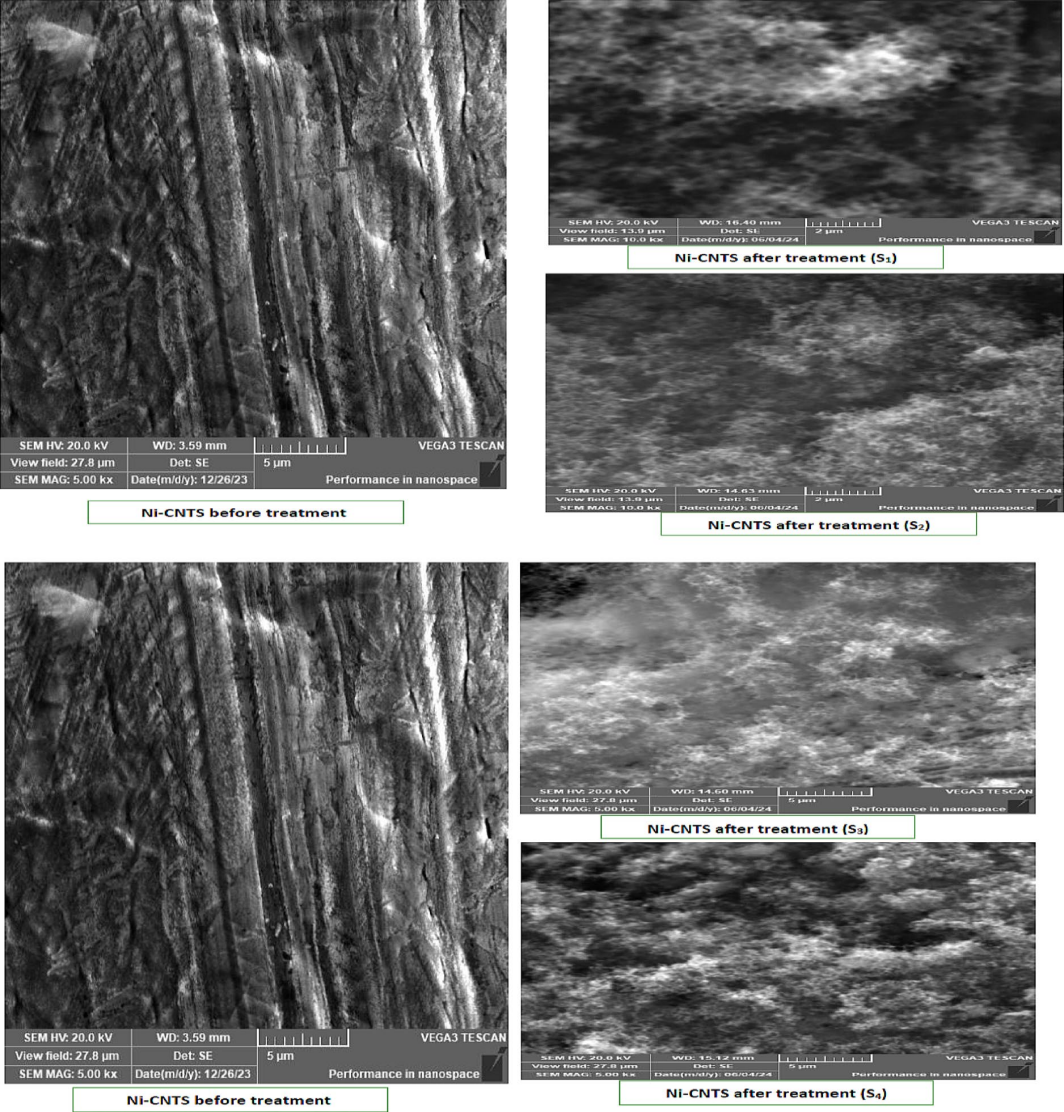
SEM for AC-MgO before and after discs 1 and 2 filtration on tractor.

**Fig. 10:**
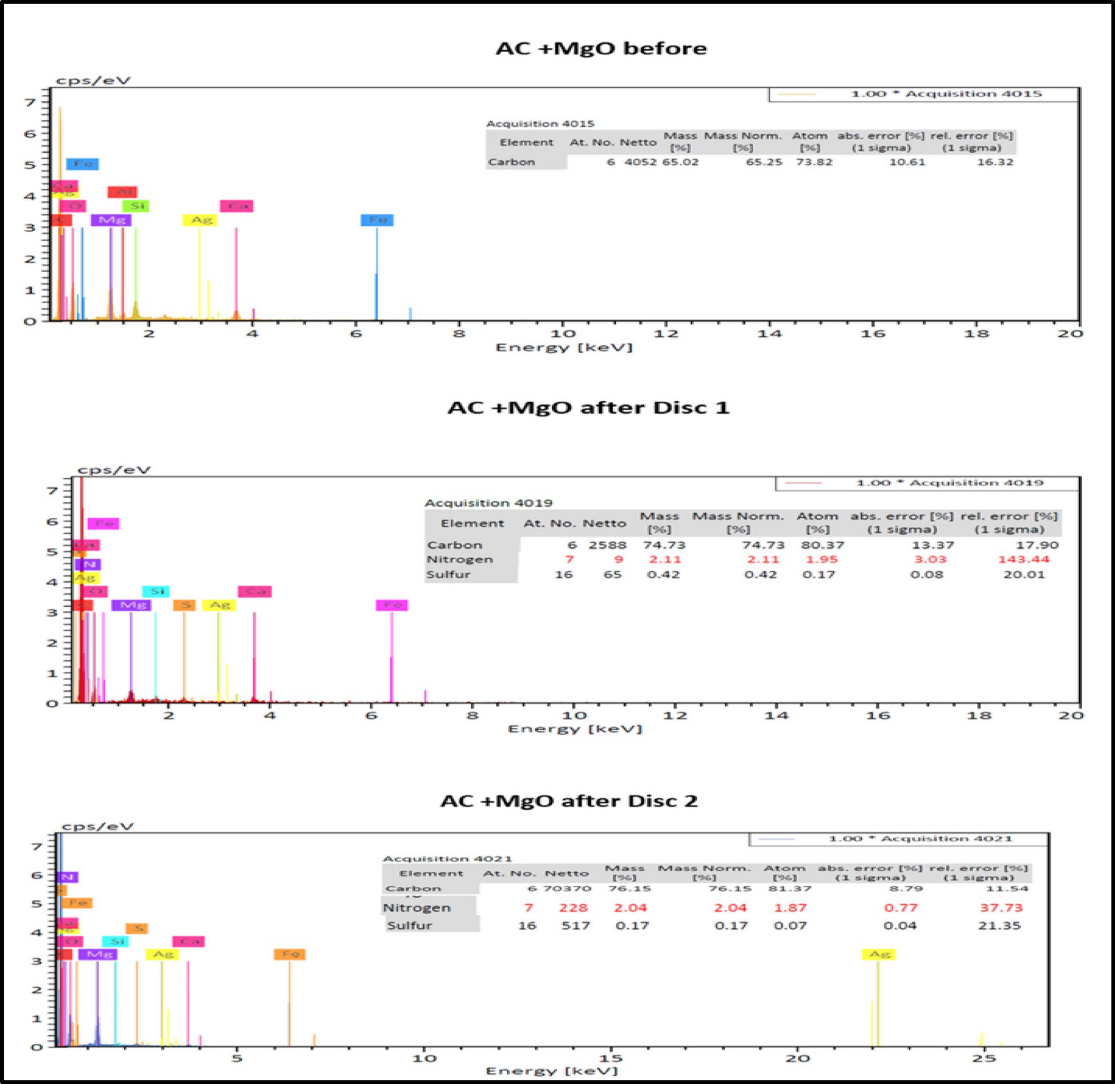
EDX for AC + MgO before and after discs 1 and 2 filtration on tractor.

SEM and EDX analyses were carried out on AC + MgO discs before and after installation. SEM images revealed structural modifications and fine particle deposition post-filtration, indicating successful pollutant capture. EDX data showed increased nitrogen, sulfur, and carbon content after use, confirming the adsorption of NO_x_, SO_2_, CO, and CO_2_. Disc 1 exhibited greater sulfur and nitrogen adsorption than Disc 2, likely due to higher exposure and greater saturation at the initial contact point. These results demonstrate the effective pollutant removal capability of MgO-enhanced AC. Figure 9 show the SME results for AC + MgO before and after discs 1 and 2 and Fig. 10 the EDX results for AC + MgO before and after discs 1 and 2. The EDX results for the activated carbon samples impregnated with magnesium oxide, both before and after filtration, are presented in Table 2. This table displays the elements and their percentages before and after the filtration process.

**Table 2:** The EDX results and the elements monitored before and after filtration on the tractor.

Element	Mess % $AC+MgO$ before	Mess % $AC+MgO$ after D1	Mess % $AC+MgO$ after D2
C	65.25	74.89	76.15
N	–	2.08	2.04
S	–	0.32	0.17

The adsorption performance of the AC + MgO unit was characterized using SEM and EDX during field operation on an M-100 Kubota tractor. SEM observations revealed noticeable structural modifications and the deposition of fine particles on the AC + MgO surface after exhaust gas exposure, indicating pollutant capture. Complementary EDX spectra confirmed increased concentrations of nitrogen, sulfur, and carbon following filtration, consistent with the adsorption of NO_x_, SO_2_, CO, and CO_2_. Notably, the first disc exhibited higher adsorption of sulfur and nitrogen species compared to the second disc, which can be attributed to its direct exposure to exhaust flow, earlier saturation of active sites, and localized chemical reactions at the surface. Collectively, these findings validate the effectiveness of the AC + MgO system in adsorbing harmful exhaust constituents under real-world tractor operating conditions.

**(b) Second prototype**

**Fig. 11:**
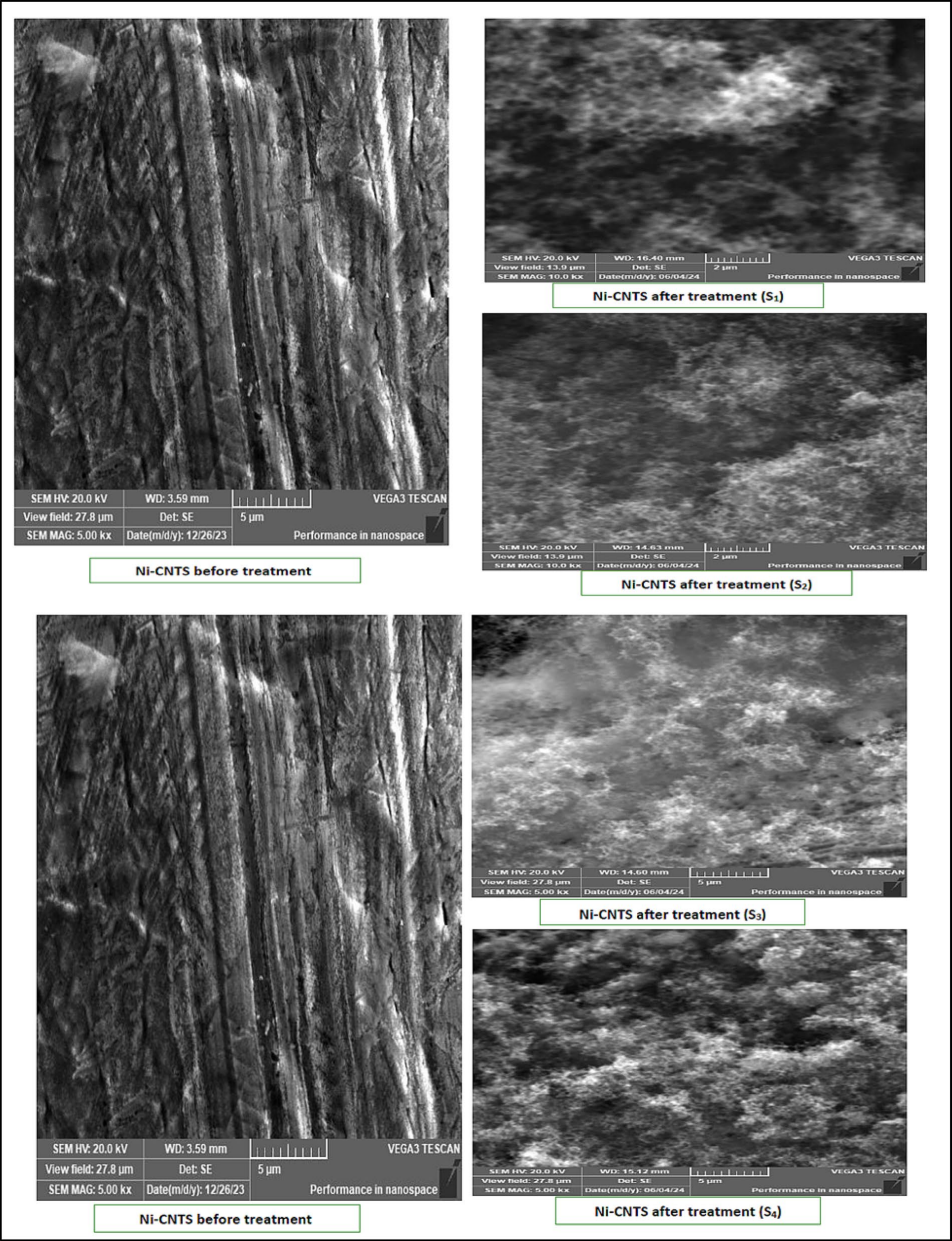
SEM for Ni-CNTS before and after filtration on tractor (S1, S2, S3 and S4).

**Fig. 12:**
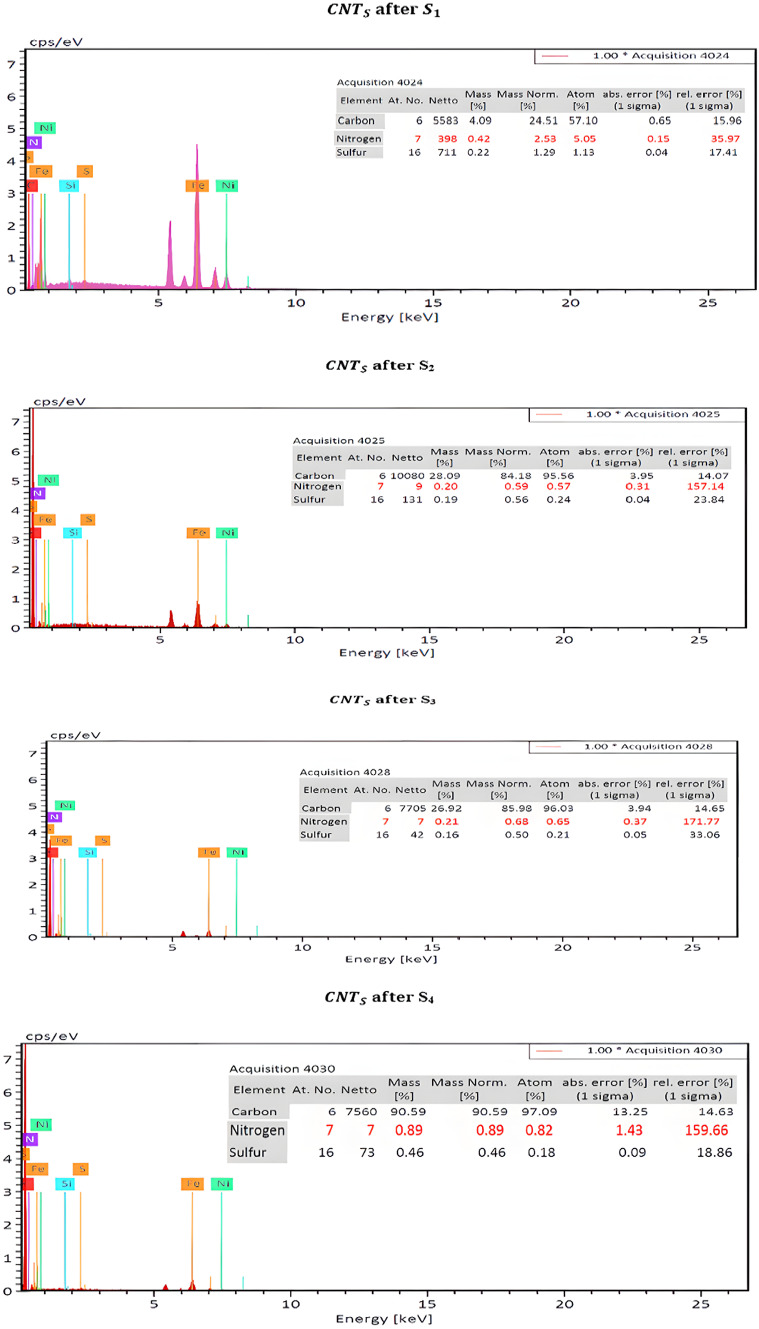
EDX for Ni-CNTS before and after and after filtration on tractor (S1, S2, S3 and S4).

SEM and EDX examinations were also performed on Ni-CNTs nanosheets before and after installation (S1–S4). Post-filtration SEM images displayed noticeable surface changes, increased surface roughness, and darkening caused by deposited exhaust particulates. EDX confirmed significant nitrogen, sulfur, and carbon accumulation, validating adsorption of NO_x_, SO_2_, and CO. The differences in pollutant capture among S1–S4 can be linked to variations in exposure time, nanoparticle distribution, and local gas flow patterns. The porous structure and uniform dispersion of CNTs likely enhanced the filtration efficiency across the nanosheets. Figure 11 show the SEM results for Ni-CNTS before and after (S1, S2, S3 and S4) and shown in Fig. 12 the EDX results Ni-CNTS before and after (S1, S2, S3 and S4).

The EDX results for the activated carbon samples impregnated with magnesium oxide, both before and after filtration, were presented in Table 3. This table displays the elements and their percentages before and after the filtration process.

**Table 3:** The EDX results and the elements monitored before and after filtration on the tractor.

Element	Ni-CNTS before	Ni-CNTS after ${S}_{1}$	Ni-CNTS after ${S}_{2}$	Ni-CNTS after ${S}_{3}$	Ni-CNTS after ${S}_{4}$
C	6.01	24.51	84.18	85.98	90.59
N	–	2.53	0.59	0.68	0.89
S	–	1.90	0.56	0.50	0.46

The adsorption behavior of the nanopaper was evaluated using SEM and EDX following exhaust gas filtration on a Kubota tractor equipped with four nanosheets. SEM analysis revealed pronounced surface modifications, including deposition-induced darkening and an apparent increase in effective surface area, indicating substantial adsorption activity. EDX spectra confirmed the presence of nitrogen, sulfur, and carbon on the nanosheets, verifying the capture of pollutants such as NO_x_, SO_2_, and CO. These results suggest that adsorption was enhanced not only by physical trapping but also through chemical interactions between the pollutants and the nanosheet surface. The overall performance was influenced by nanoparticle distribution, porosity, material density, and operating conditions, underscoring the nanosheets’ suitability for air filtration and diesel emission reduction.

### Thermal analysis on the prototypes to measure the exhaust temperature

**(a) First prototype**

The AC + MgO unit lowered exhaust gas temperature by an average of 15 °C (6.09%). Heat loss was estimated at approximately 577.5 W, and the convective heat transfer coefficient at 138.2 W/m^2^ K. This cooling effect results from the material’s high thermal capacity, porous structure, and ability to redistribute heat through gas adsorption and surface dissipation.

**1. Heat loss calculation**

The total heat loss due to the filter was estimated using the fundamental heat transfer equation:

3 $${\text{Q}} = {\text{m C}}_{{\text{P}}} \Delta {\text{T}}$$

where Q = heat loss (W), m = exhaust gas mass flow rate (kg/s), assumed as 0.035 kg/s, Cp = specific heat capacity of exhaust gases, 1100 J/kg K, ΔT = temperature drop across the filter (15 K).

By substituting the values:

$${\text{Q}} = \left( {0.0{35}} \right) \times \left( {{11}00} \right) \times \left( {{15}} \right) = {577}.{5} {\text{W}} \approx 0.{58} {\text{kW}}$$

Thus, the thermal energy dissipated due to the presence of the filtration unit is approximately 577.5 W.

**2. Convective heat transfer coefficient calculation**

The convective heat transfer coefficient (h) was determined using the following equation:

4 $${\text{Q}} = {\text{hA}}\left( {{\text{Tg}} - {\text{Ts}}} \right)$$

where h = heat transfer coefficient (W/m^2^ K)), A = surface area of the filtration unit (m^2^), estimated as 0.0628 m^2^, Tg = average exhaust gas temperature before filtration (519.5 K), Ts = estimated surface temperature of the filter (453 K).

Rearranging from Eq. (4) for h:

$$h = \frac{{\text{Q}}}{{{\text{A}}\left( {{\text{Tg}} - {\text{Ts}}} \right)}}$$

Substituting the values:

$$\begin{aligned} h & = \frac{577.5}{{0.0628 \times \left( {519.5 - 453} \right)}} \\ & = \frac{577.5}{{4.18}} = \approx 138.2\;{\text{W/m}}^{{2}} \;{\text{K}} \\ \end{aligned}$$

The AC–MgO filtration unit demonstrated a substantial cooling effect, reducing exhaust gas temperature by an average of 15 °C, which corresponds to an estimated heat loss of 577.5 W. This performance is primarily attributed to the high thermal absorption capacity of activated carbon and magnesium oxide, which not only adsorb exhaust gases but also facilitate energy redistribution within the system. The porous structure of the unit further enhances heat dissipation, supporting efficient thermal management. The convective heat transfer coefficient, measured at 138.2 W/m^2^ K, highlights the strong synergy between material properties and structural design in optimizing thermal performance^26^.

**(b) Second prototype**

The Ni-CNTs unit reduced exhaust temperature by an average of 12 °C (5.19%), corresponding to a heat loss of about 462 W and a convective heat transfer coefficient of 110.5 W/m^2^·K. CNTs’ high thermal conductivity, coupled with their adsorption properties, contributed to this cooling effect, although slightly less pronounced than in the AC + MgO unit, possibly due to differences in material density and surface morphology.

**1. Heat loss calculation**

The total heat loss due to the nano-CNT filter was estimated using the fundamental heat transfer equation:

$${\text{Q}} = {\text{m C}}_{{\text{P}}} \Delta {\text{T}}$$

where Q = heat loss (W), m = 0.035 kg/s, Cp = 1100 J/kg·K, ΔT = (12 K).

By substituting the values:

$${\text{Q}} = \left( {0.0{35}} \right) \times \left( {{11}00} \right) \times \left( {{12}} \right) = {462} {\text{W}} \approx 0.{46} {\text{kW}}$$

Thus, the thermal energy dissipated due to the presence of the filtration unit is approximately 462 W.

**2. Convective heat transfer coefficient calculation**

The convective heat transfer coefficient (h) was determined using the equation:

$${\text{Q}} = {\text{hA}}\left( {{\text{Tg}} - {\text{Ts}}} \right)$$

where A = 0.0628 m^2^, Tg = (519.5 K), Ts = (453 K).

Rearranging from Eq. (4) for h:

$$h = \frac{{\text{Q}}}{{{\text{A}}\left( {{\text{Tg}} - {\text{Ts}}} \right)}}$$

Substituting the values:

$$\begin{aligned} h & = \frac{462}{{0.0628 \times \left( {519.5 - 453} \right)}} \\ & = \frac{462}{{4.18}} = \approx 110.5\;{\text{W/m}}^{{2}} \;{\text{K}} \\ \end{aligned}$$

The Ni–CNTs filtration unit demonstrated a notable cooling effect, reducing exhaust gas temperature by an average of 12 °C and corresponding to an estimated heat loss of 462 W. This reduction is attributed to the high thermal conductivity and adsorption capacity of the carbon nanotubes, which redistribute thermal energy and enhance dissipation. The unit’s structural design and large surface area further supported efficient thermal management. The measured convective heat transfer coefficient of 110.5 W/m^2^ K indicates effective heat dissipation, although it is slightly lower than that of the AC–MgO unit, likely due to intrinsic differences in material properties and structural configuration^27^.

### Cost study

A cost assessment compared both prototypes. Prototype 1 (AC + MgO) had lower total costs (2102.5 LE/41.61 USD) than Prototype 2 (Ni-CNTs) (2730 LE/54.03 USD), primarily due to the higher price of NM_S_ used in the second design and the larger size of its filtration discs. To evaluate cost-effectiveness, a “cost per mg of NO_x_ removed” metric was calculated, showing that Prototype 1 achieved lower removal costs, highlighting its economic advantage despite Prototype 2’s slightly higher pollutant removal efficiencies. Table 4 below presents a comprehensive comparison of the costs for each prototypes.

**Table 4:** Operating costs for prototypes on tractor.

Parameter	First prototype (AC + MgO)	Second prototype (Ni-CNTs)
Fixed costs		
Manufacturing Cost of Filtration Unit	1200 LE (23.75 USD)	1200 LE (23.75 USD)
Manufacturing Cost of Two Disks	540 LE (10.69 USD)	680 LE (13.46 USD)
Cost of Stainless Steel Sheet (Quarter m^2^)	200 LE (3.96 USD)	200 LE (3.96 USD)
Total Fixed Costs	1940 LE (38.39 USD)	2080 LE( 41.16 USD)
Variable costs		
Cost of Activated Carbon/CNTs	132.5 LE (2.62 USD)	500 LE(9.90 USD)
Cost of Magnesium Oxides/Chemicals	30 LE (0.59 USD)	150LE (2.97 USD)
Total Variable Costs	162.5 LE (3.22 USD)	650 LE (12.86 USD)
Total cost	2102.5 LE (41.61 USD)	2730 LE (54.03 USD)

As shown in Table 4, the cost of the prototype one is lower than the cost of the prototype two. This is due to the fact that the NM used in the second prototype are more expensive than those used in the first prototype. Additionally, the size and cost of the disc used in the second prototype were larger than those in the first prototype.

In addition to removal efficiencies, a qualitative cost–benefit assessment was conducted to compare the economic feasibility of both prototypes. While the Ni–CNTs unit achieved marginally higher pollutant removal rates—particularly for NO_x_ and SO_2_—its material and fabrication costs were significantly higher due to the use of NM_S_ and specialized coating processes. Conversely, the AC–MgO unit, despite slightly lower removal efficiencies, demonstrated a more favorable cost-to-performance ratio, offering substantial pollutant reduction at a fraction of the cost. These findings highlight the importance of balancing technological performance with economic considerations when selecting emission control systems for agricultural machinery.

## Conclusion

Under real field conditions, the AC–MgO filtration unit effectively reduced CO, HC, NO_x_, and SO_2_ emissions from agricultural tractors, with notable performance enhancements when MgO was incorporated. The Ni–CNTs unit demonstrated superior results, achieving up to 90.8% NO_x_ and 76.1% SO_2_ reduction, alongside high removal efficiencies for CO, HC, and TSP. The superior thermal and chemical performance of the Ni–CNTs unit substantiates its applicability in next-generation emission control systems. These findings suggest that nanomaterial-based filters could be integrated into regulatory frameworks to promote sustainable agricultural machinery. Future research should focus on evaluating long-term durability, optimal cleaning intervals, and scaling the technology for commercial tractor fleets to ensure widespread adoption and sustained environmental benefits.

## Data Availability

The data that support the findings of this study are available from the corresponding author.
